# Population genetics and evolutionary history of the intertidal brittle star *Ophiothrix (Ophiothrix) exigua* in the northern China Sea

**DOI:** 10.1002/ece3.70284

**Published:** 2024-09-16

**Authors:** Qian Zhang, Xuying Hu, Zongjing Deng, Yixuan Li, Yue Dong, Chen Han, Xiaoqi Zeng, Ning Xiao, Xuelei Zhang, Qinzeng Xu

**Affiliations:** ^1^ Key Laboratory of Marine Eco‐Environmental Science and Technology, First Institute of Oceanography MNR Qingdao China; ^2^ Laboratory for Marine Ecology and Environmental Science, Qingdao Marine Science and Technology Center Qingdao China; ^3^ National Museum of Nature and Science Taito‐ku Japan; ^4^ Department of Biological Sciences, Graduate School of Science The University of Tokyo Bunkyo‐ku Japan; ^5^ Department of Biology Hong Kong Baptist University Hong Kong China; ^6^ College of Environmental Science and Engineering Ocean University of China Qingdao China; ^7^ School of Ocean Sciences, China University of Geosciences Beijing China; ^8^ Institute of Evolution & Marine Biodiversity Ocean University of China Qingdao China; ^9^ Institute of Oceanology, Department of Marine Organism Taxonomy and Phylogeny Chinese Academy of Sciences Qingdao China

**Keywords:** demographic expansion, genetic diversity, northwestern pacific, ophiuroid, population genetic structure

## Abstract

*Ophiothrix (Ophiothrix) exigua* is a common brittle star in the northwestern Pacific. As a dominant species, *O. exigua* inhabiting the intertidal rocky ecosystem are affected by multiple environmental stressors, but molecular insights into their genetic population structure remain poorly studied. In this study, we investigated the population genetics and evolutionary history of six *O. exigua* populations from the northern China Sea using mitochondrial (*COI*, *NAD4*) and nuclear (*ITS2*, *18S*) gene markers. High haplotype diversity, low nucleotide diversity, and low rates of gene differentiation among the populations of *O. exigua* were detected. Pairwise genetic differentiation (Φ_ST_) statistics between different localities were negative or low and insignificant, suggesting strong gene flow of this species over the study areas. The phylogenetic analyses showed that the populations exhibited high homogeneity between localities in our study area. Demographic analyses indicated that the populations experienced sustained expansion around 0.2 million years ago. This expansion was likely related to transgressions events in the Yellow Sea during the Pleistocene period. Additional samples of *O. exigua* from disparate geographical locations, especially the Japan Sea and the Korean Peninsula, will be needed to unravel the population genetic patterns and evolutionary history of this species.

## Graphical abstract

This study investigated the genetic diversity, population structure, and demographic history of the brittle star Ophiothrix (Ophiothrix) exigua, a common intertidal species in the northwestern Pacific Ocean, using mitochondrial and nuclear gene markers. The results revealed high genetic diversity but remarkably low genetic differentiation among populations in the Yellow and Bohai Seas, suggesting widespread gene flow and the absence of cryptic speciation within the study area. Demographic analyses indicated that the populations experienced expansion around 0.2 million years ago, likely related to sea level changes during the Pleistocene glaciations.

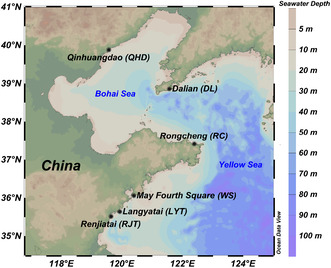

## 1 INTRODUCTION

Genetic diversity is a vital component of biodiversity and a key parameter for assessing the status of biological resources (Cen et al., 2023). Rich genetic diversity helps enhance the adaptability and evolutionary potential of species (Hughes et al., 2008; Yang et al., 2016). Various processes such as divergence, isolation, and bottleneck all influence the levels of genetic diversity of population (Raman et al., 2023). However, the genetic diversity, population structure, and ecological functions of marine organisms have all been profoundly affected by global warming and human activities (Donelson et al., 2019; Eirin‐Lopez & Putnam, 2019; Törnroos et al., 2019). Rising ocean temperatures pose a significant challenge to the survival of marine organisms (Schiel et al., 2004), potentially leading to the northward migration of some benthic organisms (Xu et al., 2023). Assessing and maintaining the genetic diversity of marine organisms is crucial for preserving biodiversity and sustaining the health of marine ecosystems.

The Yellow and Bohai Seas, as important continental marginal seas in the northwestern Pacific Ocean (Liu, 2013), are sensitive to global change due to the wide, flat, and low‐gradient shelves (Liu et al., 2016, 2018). Since the late Quaternary, the frequent “transgression” and “regression” events in the Yellow and Bohai Seas have been primarily driven by the significant fluctuations in sea level caused by climate change (Liu et al., 2014; Yao et al., 2017). Sedimentary environment studies in the Yellow and Bohai Sea region have revealed that this area has experienced at least four marine transgression events since 200 Ka (Yao et al., 2022), which likely correspond to the interglacial periods indicated by Marine Isotope Stages (MIS)7, 5, 3, and 1 (Yao et al., 2022; Yi et al., 2013). These cyclic transgression and regression events have significantly impacted the population structure of marine species inhabiting the Yellow and Bohai Sea Shelf (Avise, 2000; Han et al., 2008; Shen et al., 2011; Yang & Li, 2016), leading to population growth and range expansion (Han et al., 2008; Wang, 1999) during transgression or population bottlenecks, genetic drift, and fragmented or confined to refugia during regression (Liu et al., 2006; Voris, 2000). The alternation between transgression and regression events notably affected intertidal species, likely shaping their current distribution patterns and driving their rapid adaptation to ever‐changing environments (Xue et al., 2014).

*Ophiothrix (Ophiothrix) exigua* Layman, 1874, is a common and dominant ophiuroids in the intertidal zone (Liao, 2004; Yi & Irimura, 1987) and is reported from the surrounding intertidal areas of Qingdao Coast (Liao, 2004), East Sea, Chindo & Cheju Island of Korean peninsula (Shin, 1995), Bousou peninsula, Inland Sea, Japan Sea (Kitazawa et al., 2015). As the ecological keystone species, *O. exigua* plays crucial roles in ecosystems, making significant contributions to the marine carbon cycle and calcium reservoirs (Johnston & Gruner, 2018; Lebrato et al., 2010; Lessin et al., 2019). *Ophiothrix (Ophiothrix) exigua* is also sensitive to temperature fluctuations, making it a good bioindicator of ecological stress (Fuad et al., 2022; Wang et al., 2023). However, *O. exigua* may be a cryptic species complex rather than a single species due to its highly diverse morphology, indicating various colors (gray, yellow, and brown), patterning of arms, and arrangement of spinelets around the disk (Boissin et al., 2008; Hernández‐Díaz et al., 2023; Taboada & Pérez‐Portela, 2016). That may bring difficulties to its identification (Leiva et al., 2023). Such morphological variants were subsequently made this species improperly identified as several species, for instance, *O. hylodes* H.L. Clark, 1911, *O. marenzelleri* Koehler, 1904, and *O. stelligera* Lyman, 1874. They were recently proved synonymised with *O. exigua* (Irimura, 1981). Furthermore, this species exhibits a high reproductive capacity, developing from small eggs into juvenile stages within approximately 1 month through a planktotrophic larva (Kitazawa et al., 2015).

In this study, we aim to elucidate the population genetics and evolutionary history of *O. exigua* from the northern China Sea using DNA sequence data from both mitochondrial and nuclear genes to (1) assess the genetic structure among different geographic populations; (2) examine the genetic diversity; and (3) infer the demographic changes of *O. exigua*.

## 2 MATERIALS AND METHODS

### 2.1 Sampling and DNA extraction

**FIGURE 1:**
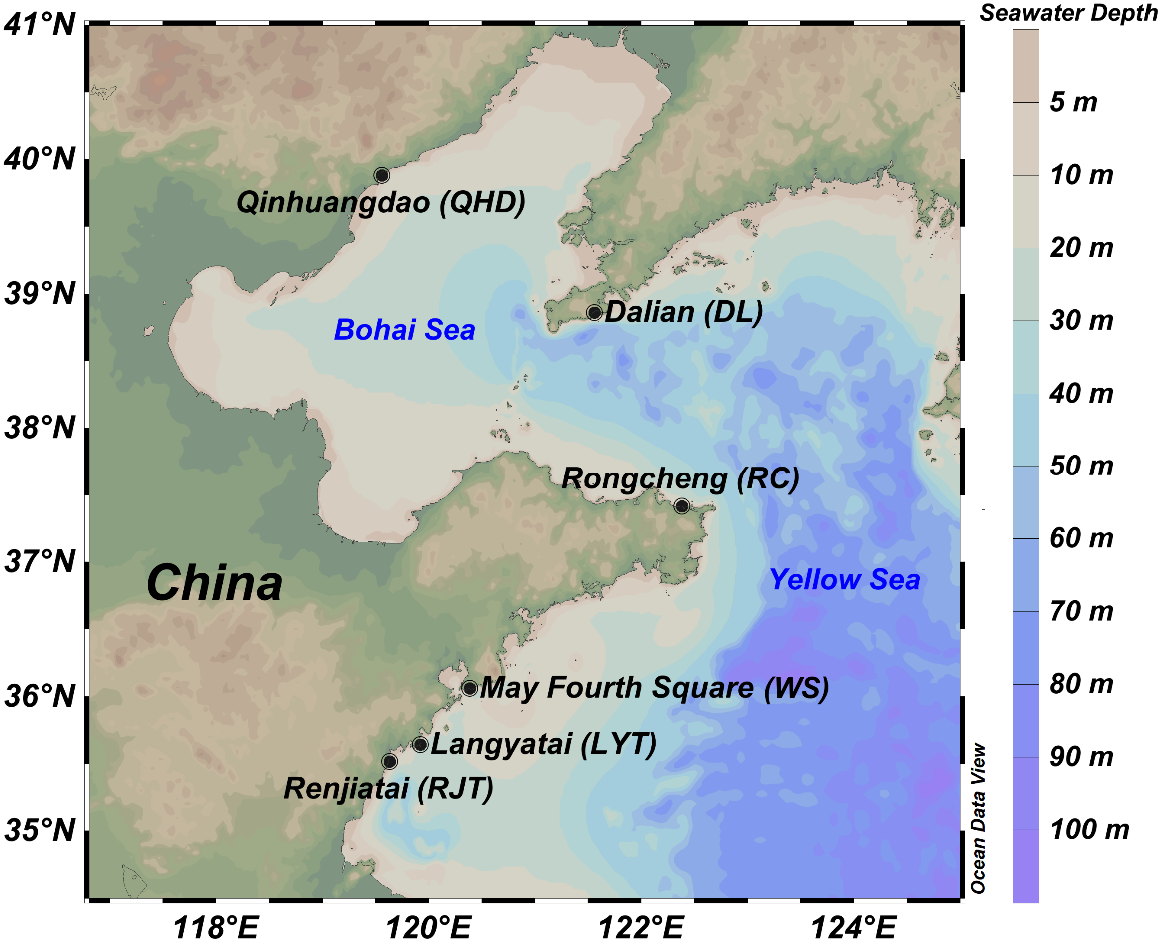
Distribution map showing sampling locations of *Ophiothrix (Ophiothrix) exigua* populations. This map was made via Ocean Data View software. (https://odv.awi.de).

A total of 80 samples of *O. exigua* were collected from six intertidal zones of locations including Qinhuangdao (QHD), Dalian (DL), Rongcheng (RC), Langyatai (LYT), Renjiatai (RJT), and May Fourth Square (WS, Figure 1). The morphology of *O. exigua* exhibits slight variation between different sampling points (Table S1). After morphological identification, the collected samples were fixed with absolute ethanol and stored at −20°C. A segment of approximately 1 cm from the arm tissue of the brittle star was taken for DNA extraction. After grinding in liquid nitrogen, total genomic DNA was extracted using either the QIAamp Fast DNA Tissue Kit (QIAGEN, Germany) or the OMEGA Tissue DNA Kit (OMEGA, USA), following the specific extraction procedures provided in the respective manuals. The extracted total genomic DNA was qualified by 1% agarose gel electrophoresis before PCR amplification.

### 2.2 PCR amplification and sequencing

For each sample, we conducted amplification with specific primers for two mitochondrial genes—cytochrome c oxidase subunit I (*COI*) and NADH dehydrogenase subunits 4 (*NAD4*)—and two nuclear genes—Internal Transcribed Spacer 2 (*ITS2*) and 18S ribosomal RNA (*18S*) (Table 1). The primers for the *NAD4* were designed based on the complete mitochondrial genome sequence of *O. exigua* (GenBank number: ON729296) (Table 1). PCRs were conducted in a total volume of 25 μL mixture, comprising 1 μL of each primer (10 μM), 9.5 μL of diluted distilled H_2_O, 12.5 μL of 2× Taq PCR Master Mix (with blue dye, Sangon Biotech, Inc), and 1 μL of the template DNA. The PCR cycling conditions for *COI*, *ITS2*, and *18S* were followed as described in the respective references (Table 1). The amplification of *NAD4* used the following profile: The first step was at 95°C for 3 min, followed by 35 cycles at 95°C for 30 s, 50°C for 30 s and 72°C for 1 min, and a further extension at 72°C for 10 min. The PCR products were sequenced for both directions by Sangon Biotech, Inc. (Qingdao, China) with the same primers used in the amplification reaction. All sequences of this study were uploaded to the GenBank database (Table 2).

**TABLE 1:** The PCR primers for amplification of *COI*, *NAD4*, *18S* and *ITS2* sequences.

Destination fragment	Primer name	Primer sequence (5′‐3′)
*COI* (Folmer et al., 1994)	COI‐F	TTCTACCAACCATAAGGATATAGGG
	COI‐R	TTCTACCAACCATAA GGATATAGGG
*NAD4* (This study)	NAD4‐F	CTACCACAGCCGCCATTA
	NAD4‐R	TCCGTATCCTCCCATCTT
*18S* (Hunter et al., 2016)	18F997	TTCGAAGACGATCAGATACCG
	18R1779	TGTTACGACTTTTACTTCCTCTA
*ITS2* (Naughton et al., 2014)	OphITS2F	TCGTTCGTCTGAGGGTCGTTA
	OphITS2R	GTAGTCTCGCTCGATCTG

**TABLE 2:** The information of GenBank accession.

Locus	GenBank accession number
*COI*	OR782206‐OR782277, OR984206‐OR984213
*NAD4*	OR780679‐OR780743, PP001162‐PP001168
*ITS2*	OR784232‐OR784298, PP001711‐PP001718
*18S*	OR775595‐OR775657, OR988137‐OR988143

### 2.3 Data analysis

The sequenced fragments obtained from bidirectional sequencing were assembled using Dnastar Lasergene v7.1 (Clewley, 1995). All sequences were checked and aligned using the ClustalW algorithm (Thompson et al., 1994) available in MEGA v7 (Kumar et al., 2016). The alignment of each gene fragment was then auto‐trimmed by G‐blocks (Jose, 2000) with default settings to output conserved datasets. Genetic diversity indices, including the number of nucleotide composition, segregating sites (S), number of haplotypes (H), nucleotide diversity (π), haplotype diversity (Hd), and gene flow, were estimated for each dataset using DnaSP v6.12.03 (Librado & Rozas, 2009). The 18S dataset showed highly conserved intraspecific variation, with populations from the six locations sharing a single haplotype. Due to the lack of informative variation, the 18S marker was excluded from further analyses.

To determine the evolutionary relationships among haplotypes, we constructed phylogenetic trees using the Bayesian Inference (BI) and maximum likelihood (ML) methods. Using PAUP 4.0b10 (Swofford, 2002), the partition homogeneity test revealed no significant incongruence among the *COI*, *NAD4*, and ITS2 datasets (*p* = .3200), allowing them to be combined for phylogenetic analysis. We determined the best‐fit model (HKY + G) for BI and ML analyses using ModelFinder v1.5.4 (Kalyaanamoorthy et al., 2017) embedded in IQ‐TREE v1.6 (Nguyen et al., 2015). We used *Ophiothrix (Acanthophiothrix) scotiosa* Murakami, 1943, and *Ophiothrix (Acanthophiothrix) proteus* Koehler, 1905, as the outgroups. The ML analysis of concatenated *COI*‐*NAD4*‐*ITS2* sequence datasets was carried out using IQ‐TREE with 10,000 ultrafast bootstrap (Hoang et al., 2018). BI analysis was implemented using MrBayes v3.2.6 (Ronquist et al., 2012) on two separate runs of Markov chains for 1 million generations with the first 25% of trees as burn‐in. Effective sample size (ESS) was determined using Tracer v1.7 (Rambaut et al., 2018) to ensure all parameter values with ESS >200. Based on the phylogenetic analyses and population delimitation, pairwise genetic distances were computed in MEGA using the Kimura 2‐parameter (K2P) model (Kimura, 1980).

Genetic differentiation index (Φ_ST_) and analysis of molecular variance (AMOVA) among geographic populations were analyzed using Arlequin v3.5.2.2 (Excoffier & Lischer, 2010), testing the distribution frequency differences of haplotypes among populations. The Φ_ST_ values were estimated using a pairwise difference distance matrix, and significance levels were assessed by 1000 permutations. Tajima'D (Tajima, 1989) and Fu's Fs (Fu, 1996) neutrality tests were used to infer the historical demography of *O. exigua* populations. A TCS haplotype network (Clement et al., 2002) was constructed using POPART v.1.7 (Leigh & Bryant, 2015) to determine the genealogical relationships between haplotypes, visualizing the overall structure and spatial pattern of genetic diversity from the haplotype data of *O. exigua*.

The mismatch distribution of populations from different locations was estimated by DnaSP to test the frequency distribution of pairwise differences under past population change. The demographic parameter tau (τ) was determined using DnaSP software to estimate the time since regional population expansion. The equation *t* = 2μk/τ (Rogers & Harpending, 1992) was used to determine the population size change of *O. exigua*, where t is the number of years since population expansion, μ is the per site per year substitution rate, and k refers to the gene fragment size. Finally, to assess changes in the size of the *O. exigua* population over time, we plotted the Bayesian Skyline Plots (BSP) using BEAUti v 2 and BEAST v 2.6.3 (Bouckaert et al., 2014). For both mismatch and BSP analyses, a substitution rate of 2.48 × 10^−8^ per site per year was utilized for the *COI*, in accordance with previous studies in Ophiuroidea (Naughton et al., 2014).

## 3 RESULTS

### 3.1 Sequence characterization

A total of 80 *COI*, 72 *NAD4*, 75 *ITS2*, and 70 *18S* sequences were successfully amplified for 80 individuals. The average sizes of *COI*, *NAD4*, *ITS2*, and *18S* gene fragments were 584, 645, 586, and 731 bp, respectively. All four gene sequences showed a high similarity (>98.97%) to the complete mitochondrial genome sequence of *O. exigua* in GenBank (ON729296). The average frequencies of the four nucleotides in the *COI* and *NAD4* gene regions for all samples of *O. exigua* were 54.54% (A + T), 45.46% (G + C), 61.70% (A + T), and 38.30% (G + C), respectively. The A + T content was higher than the G + C content, which was consistent with the characteristics of the mitochondrial base composition (Galaska et al., 2019; Scouras et al., 2004; Sun et al., 2023).

### 3.2 Genetic variation

We calculated genetic diversity statistics for the six locations of *O. exigua* using three gene fragments (Table 3). The populations presented high haplotype diversity, with Hd values ranging from 0.995 to 0.998, and low nucleotide diversity, with π values between 0.00882 and 0.01372. *NAD4* exhibited the highest nucleotide diversity (π = 0.01372), followed by *COI* (π = 0.00985). In contrast, *ITS2* appeared to be the most conserved gene in *O. exigua* compared with the other two fragments (π = 0.00032). Six populations all presented high genetic diversity according to their high haplotype diversities (Hd: 0.933–1). The nucleotide diversities ranged from 0.00760 to 0.01622, with the LYT population exhibiting the highest values for both single mitochondrial gene (π = 0.01119 for *COI*, π = 0.01622 for *NAD4*) and the concatenated genes (π = 0.01074).

**TABLE 3:** Genetic diversity statistics of *Ophiothrix (Ophiothrix) exigua* populations.

Locus	Location	*N*	S	H	π	Hd	Tajima's D	Fu's fs
*COI*	QHD	15	30	15	0.01001	1.000	−1.54117	−10.85574**
	DL	11	28	11	0.00996	1.000	−1.80781*	−6.31013*
	RC	20	40	20	0.01078	1.000	−1.75973*	−16.86731**
	WS	12	25	12	0.00760	1.000	−2.06612**	−8.85344**
	LYT	10	31	8	0.01119	0.933	−1.94261*	−1.29468
	RJT	12	24	11	0.00905	0.985	−1.48734	−5.26936*
	Total	80	95	71	0.00985	0.995	−2.39742**	−8.85344*
*NAD4*	QHD	15	44	15	0.01574	1.000	−1.07019	−7.36020^**^
	DL	11	32	11	0.01161	1.000	−1.46104	−5.24451**
	RC	14	39	14	0.01481	1.000	−0.96158	−6.79857**
	WS	12	33	11	0.01200	0.985	−1.31715	−3.72781*
	LYT	8	34	8	0.01622	1.000	−1.07534	−2.08024
	RJT	12	31	10	0.01250	0.970	−0.87725	−2.17401
	Total	72	84	65	0.01372	0.997	−1.77614	−4.56422**
*ITS2*	QHD	15	2	2	0.00046	0.133	−1.49051	−0.23493
	DL	11	0	1	0.00000	0.000	0.00000	0.00000
	RC	16	2	2	0.00043	0.125	−1.49796	−0.17695
	WS	12	2	3	0.00057	0.318	−1.45138	−1.32484^*^
	LYT	9	0	1	0.00000	0.000	0.00000	0.00000
	RJT	12	1	2	0.00028	0.167	−1.14053	−0.47566*
	Total	75	6	6	0.00032	0.130	−2.00119^*^	−6.66178*
*COI + NAD4* + *ITS2*	QHD	15	73	15	0.00956	1.000	−1.49051	−5.38584**
	DL	11	60	11	0.00838	1.000	−1.66233*	−3.30375*
	RC	14	61	14	0.00837	1.000	−1.35124	−5.26169*
	WS	12	58	12	0.00767	1.000	−1.68562*	−4.21960*
	LYT	8	59	8	0.01074	1.000	−1.34847	−1.17196
	RJT	12	54	12	0.00839	1.000	−1.16998	−3.91670*
	Total	72	170	69	0.00882	0.998	−2.14571*	−3.41603**

Abbreviations: DL, Dalian; H, number of haplotypes; Hd, haplotype diversity; LYT, Langyatai; N, number of sequences; QHD, Qinhuangdao; RC, Rongcheng; RJT, Renjiatai; S, number of segregating sites; WS, May Fourth Square; π, nucleotide diversity.

*p <  .05.

**p <  .01.

### 3.3 Phylogenetic and network analyses

**FIGURE 2:**
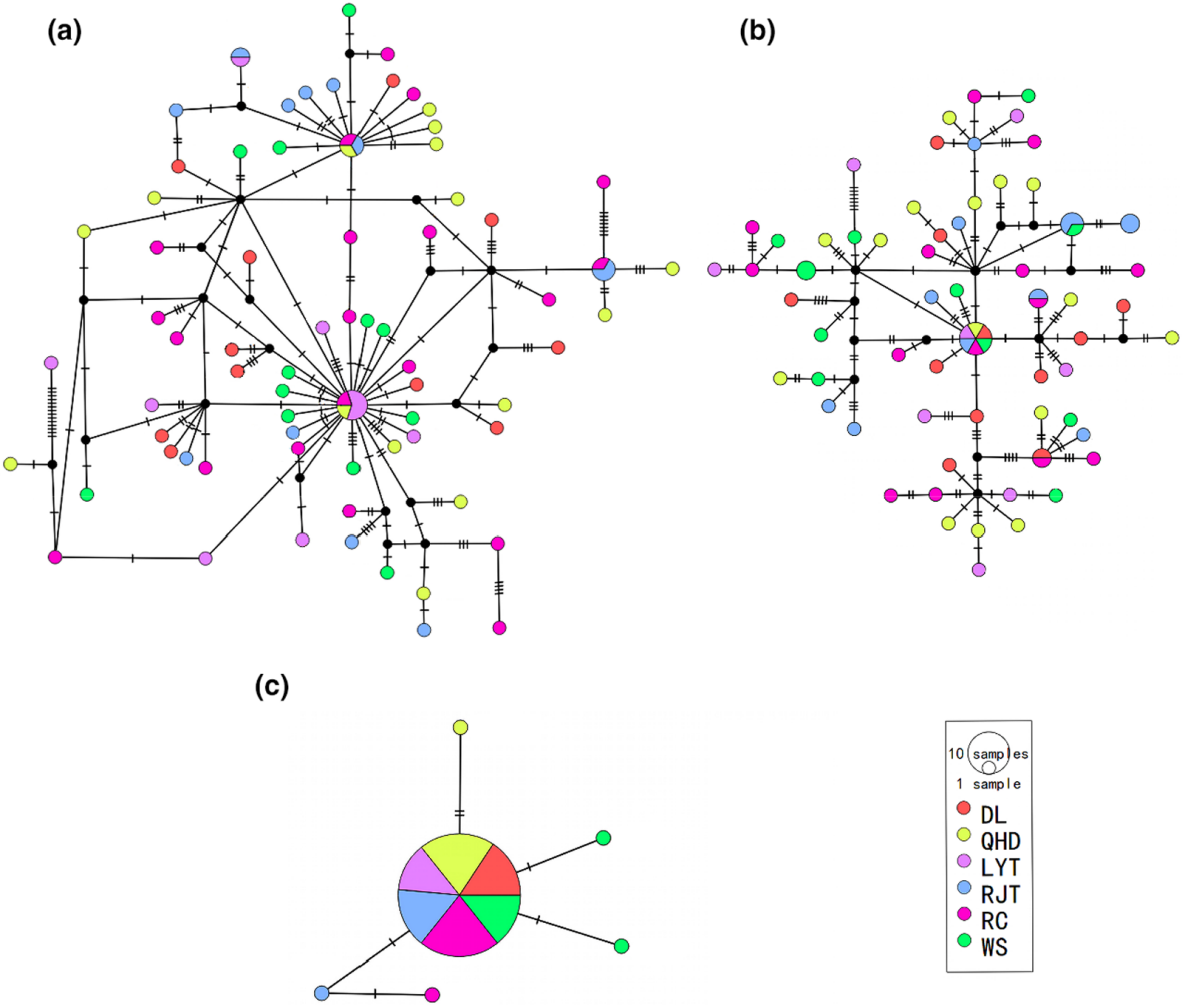
The network based on (a) *COI*, (b) *NAD4* and (c) *ITS2* haplotypes. Numbers and each circle represent a unique haplotype; size is proportional to their frequencies; colors denote populations; dashes in the branches represent the number of mutation steps. This figure was generated using POPART v.1.7. DL, Dalian; LYT, Langyatai; QHD, Qinhuangdao; RC, Rongcheng; RJT, Renjiatai; WS, May Fourth Square.

The median‐joining network (Figure 2) showed the connection among the six *O. exigua* populations. The *ITS2* network had the simplest topology, with the highest number of shared haplotypes (H1) located at the center, while other haplotypes differed by only one or two substitutions from this main haplotype. Based on coalescent theory, H1 may represent the ancestral haplotype of *O. exigua*. In contrast, the *COI* and *NAD4* networks revealed profound diverse bush‐like genealogies, with black circles indicating missing haplotypes, and numerous haplotypes broadly and evenly distributed. The haplotypes from each sampling location were homogeneously mixed in the networks, failing to show distinct geographical patterns or population differentiation.

To study the phylogenetic relationship of *O. exigua* in six regions, we constructed the haplotype phylogenetic trees using the concatenated *COI*‐*NAD4*‐*ITS2* gene set for all populations. ML and BI analyses produced phylogenetic trees with consistent topology but with different supporting values (Figure S1). The phylogenetic analysis of *O. exigua* populations revealed a lack of stable geographical differentiation. Despite the presence of unique haplotypes within each population, individuals from different locations were intermixed in the phylogenetic tree, without any obvious group clustering observed.

### 3.4 Genetic structure

The AMOVA results showed that a large proportion of molecular genetic variation was present within populations (99.25%, *p* < .001), and the remaining variation was found among populations (0.75%, *p* < .001) for the concatenated sequences. The overall fixation index (F_ST_) observed for all populations was nonsignificant (0.00753, *p* > .05, Table 4). Consistent with the concatenated dataset, the genetic variance percentages were high for all three markers: 99.94% for *COI*, 100.12% for *NAD4*, and 99.25% for *ITS2* (Table 5). Pairwise Φ_ST_ values between populations varied from −0.009 (QHD‐WS) to 0.081 (DL‐RJT) for the concatenated dataset. Population pairwise Φ_ST_ analysis among all populations demonstrated no significant genetic differentiation. The mean K2P distance was between 0.008 and 0.010 for all populations (Table 5).

**TABLE 4:** AMOVA analysis for the population of *Ophiothrix (Ophiothrix) exigua*.

Source of variation	Df	Sum of squares	Variance components	Percentage of variation (%)	F_ST_
COI					
Among populations	5	14.488	0.00175 Va	0.06	0.00061
Within populations	74	212.724	2.87465 Vb	99.94	
Total	79	227.213	2.87640		
NAD4					
Among populations	5	21.824	−0.00537 Va	−0.12	−0.00121
Within populations	66	292.301	4.42880 Vb	100.12	
Total	71	314.125	4.42343		
ITS2					
Among populations	5	0.388	−0.00132 Va	−1.43	−0.01426
Within populations	69	6.492	0.09408 Vb	101.43	
Total	74	6.880	0.09276		
COI + NAD4 + ITS2					
Among populations	5	37.955	0.05281 Va	0.75	0.00753
Within populations	66	459.462	6.96155 Vb	99.25	
Total	71	497.417	7.01436		

**TABLE 5:** Mean K2P genetic distance (below diagonal) and pairwise Φ_ST_ values (above diagonal) between populations based on the concatenated genes.

Locality	QHD	DL	RC	WS	LYT	RJT
QHD		−0.020	−0.017	−0.009	−0.028	0.052
DL	0.009		−0.013	−0.012	−0.021	0.081
RC	0.009	0.008		−0.018	−0.019	0.025
WS	0.009	0.008	0.008		−0.028	0.061
LYT	0.010	0.009	0.009	0.009		0.065
RJT	0.010	0.009	0.009	0.009	0.010	

Abbreviations: DL, Dalian; LYT, Langyatai; QHD, Qinhuangdao; RC, Rongcheng; RJT, Renjiatai; WS, May Fourth Square.

### 3.5 Historical dynamics of the population

**FIGURE 3:**
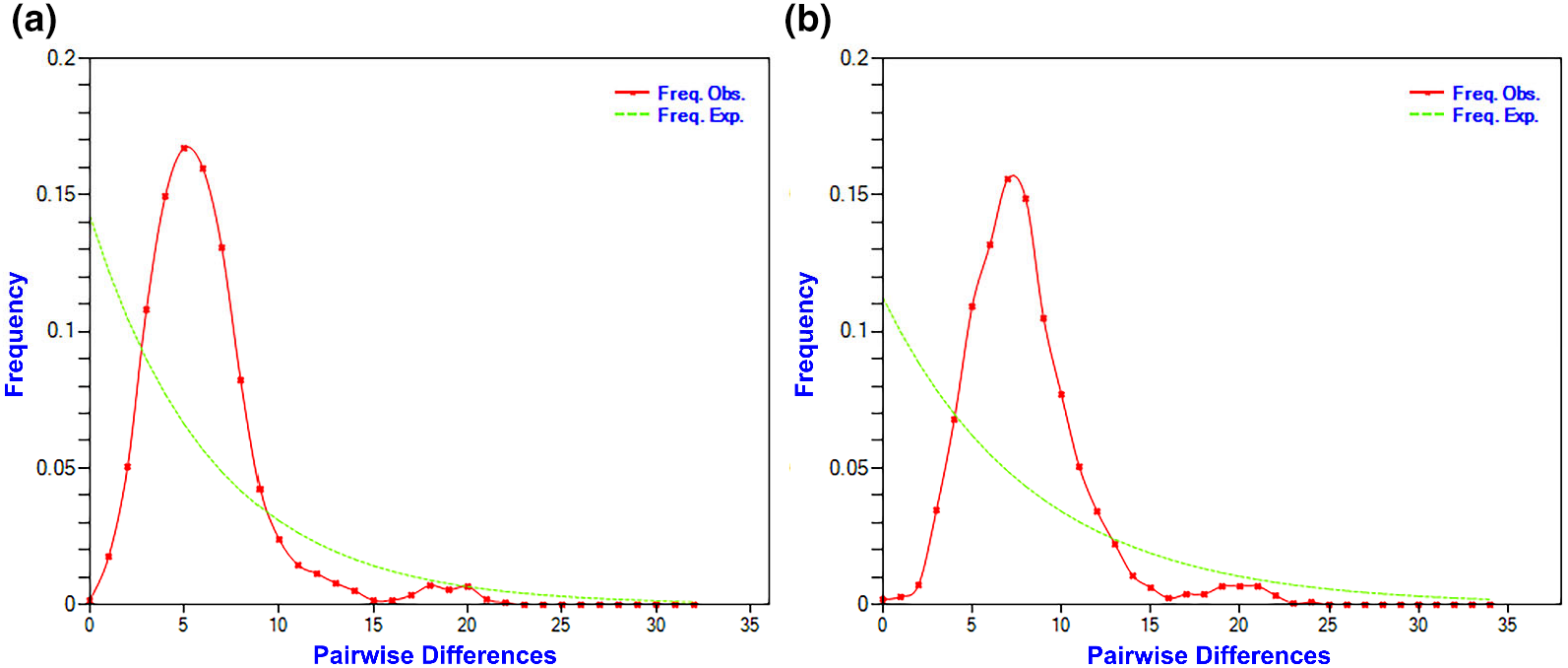
The mismatch distributions for all individuals in Northern China Sea. (a) *COI* and (b) *NAD4*. This figure was generated using DnaSP v6.12.03.

**FIGURE 4:**
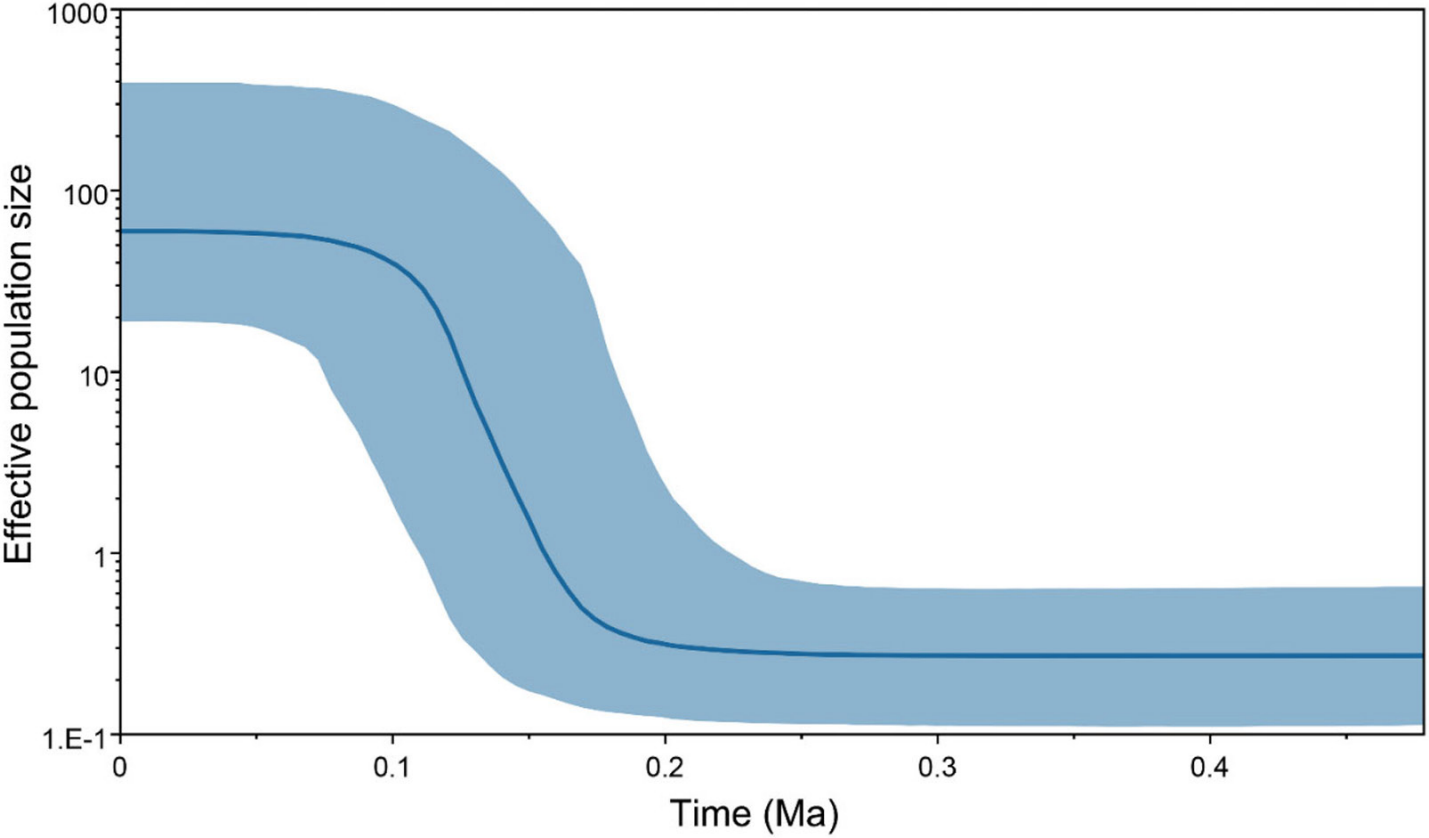
BSP Analysis of *COI* sequence datasets for all individuals in Northern China Sea. The X‐axis represents time in millions of years before the present. The Y‐axis represents the effective population size. The solid line indicates the median population size, and the 95% interval of HPD is shown in blue. This was generated using BEAUti v 2 and BEAST v 2.6.3.

BSP analysis, mismatch distribution, and neutrality tests were employed to investigate the historical demography of the *O. exigua* population. The overall negative neutrality tests (Tajima's D and Fu's Fs) suggest that *O. exigua* encountered a population bottleneck followed by expansion (Table 3). The mismatch distribution curves drawn based on *COI* and *NAD4* mitochondrial gene sequences showed unimodal distribution, which was consistent with the population expansion model (Figure 3). Using the formula *t* = 2μk/τ (τ = 5.6), the time of the expansion event was calculated as 193,327 years before present (BP). BSP analysis of the total population also showed an increase in the effective population size, indicating that the populations experienced sustained expansion at about 0.2 Ma (Figure 4) and that the population rapidly grew to its current size.

## 4 DISCUSSION

In the present study, we found high population homogeneity across the Yellow and Bohai Seas. The populations of *O. exigua* in the studied areas exhibit a relatively high level of genetic diversity, but no population divergence (Figure 2 and Table 3). Our results demonstrated that the different morphs present in *O. exigua* correspond to a single evolutionary unit, with no evidence of cryptic species, as we observed no genetic divergence was observed between those variant individuals. The homogeneity in *O. exigua* contrast with the high number of cryptic speciation events detected in other *ophiothrix*, such as *Ophiothrix fragilis f. nuda* Madsen, 1970 (Pérez‐Portela et al., 2013; Taboada & Pérez‐Portela, 2016) and *Ophiothrix angulata* Say, 1825 (Hernández‐Díaz et al., 2023). The difference in morphological variation of *O. exigua* may be the result of phenotypic plasticity due to adaptation to the environment. Furthermore, the extremely high genetic diversity found in *O. exigua* seems to be a characteristic of some abundant ophiuroids likely related to large population sizes (Hunter & Halanych, 2010), which makes populations less susceptible to genetic drift and helps maintain a high‐level of low‐frequency haplotypes within populations (Muths et al., 2009; Pérez‐Portela et al., 2013). A higher genetic diversity also helps to enhance the adaptability of *O. exigua* to the environment (Bonin et al., 2007; Cen et al., 2023).

The lack of significant differences among various geographical areas in *O. exigua* may result from the combination of life‐history traits and ocean current circulation patterns (Leiva et al., 2023; Pérez‐Portela et al., 2017; Taboada & Pérez‐Portela, 2016). One key factor is the pelagic larval duration (PLD), which has a significant impact on gene flow and genetic structure in benthic organisms with a larval stage (Scheltema, 1986, 1995; Selkoe & Toonen, 2011). The planktotrophic larva of *O. exigua* remains in the water column for approximately 1 month (Kitazawa et al., 2015), exhibiting a strong dispersal ability that allows it to cross geographical barriers, thus significantly enhancing gene exchange. Additionally, ocean currents play a crucial role in larval dispersal (Scheltema, 1986). For instance, northward currents such as the Yellow Sea Warm Current and the Liaonan Coastal Current facilitate the northward dispersal of planktonic larvae from the DL population. Meanwhile, southward currents such as the Bohai Bay Coastal Current, Liaodong Bay Coastal Current, and Lubei Coastal Current promote the southward migration of planktonic larvae from QHD and RC populations, thereby enabling gene exchange (Chen et al., 2020; Li, Yang, et al., 2020; Ramírez Ayala, ; Yang et al., 2019). Frequent gene flow leads to the homogenization of genetic resources, resulting in the absence of significant genetic differentiation and typical geographical structure among *O. exigua* populations in the northern China Sea.

The population homogeneity found in *O. exigua* could be also the result of historical recolonization events, including habitat expansion and demographic growth during the Pleistocene period. In this study, the results of haplotype diversity, the neutral theory test (Tajima's D and Fu's Fs), mismatch distribution and BSP all support the occurrence of a rapid population expansion event for *O. exigua* in the northern China Sea. The expansion time were estimated using different methods in this study. Mismatch analysis estimated the expansion time to be around 193,327 years ago, while BSP analysis dated the expansion event to 0.2–0.1 Ma. This timing closely aligns with three significant marine transgressions in the studied area since the Pleistocene. These transgressions correspond to high sea‐level periods during MIS7a (197–191 ka, an interglacial period), MIS6 (130–191 ka, possibly influenced by regional tectonic activity), and MIS5e (130–115 ka, the Eemian Interglacial) (Bintanja et al., 2002; Wang et al., 2020; Yi et al., 2012, 2013). During glacial maxima, with a sea level drop of 130–150 m, the Bohai Sea and Yellow Sea Shelf were completely exposed (Liu et al., 2007; Xie et al., 1995). Therefore, we have sound reason to infer that different geographical populations of *O. exigua* might coalesce together due to the geographical range contraction during the glacial period and then expand population range when favorable conditions emerged during interglacial periods (Xi et al., 2023). The periodic coalescence of populations could enhance historical gene flow among populations (Xue et al., 2014). Expansion events in this period were well‐documented for other dominant species in the same regions, such as *Nibea albiflora* Richardson, 1846 (170–85 ka) (Han et al., 2008), *Ophiura sarsii vadicola* Djakonov, 1954 (111.8–60.1 ka) (Li, Dong, et al., 2020), and *Atrina pectinata* Linnaeus, 1767 (ca. 160 ka) (Xue et al., 2014). The genetic population patterns of those species were similar to *O. exigua*, with high genetic diversity and low population divergence.

The natural geographic distribution of *O. exigua* includes the China Seas (Liao, 2004), the Japan Sea (Kitazawa et al., 2015; Okanishi et al., 2024), the Korean Peninsula (Shin, 1995), and other northwestern Pacific areas. Although *O. exigua* is widely distributed, this study focused solely on the northern China Sea. To unravel the population genetic patterns and evolutionary history of *O. exigua* in the northwestern Pacific areas, more samples from disparate geographical locations, especially the Japan Sea and the Korean Peninsula, need to be collected. The southern China Sea was not considered in our study because the southern specimens of this species in earlier records may have been misidentified. We confirmed significant morphological differences between our fresh *O. exigua* specimens and museum samples from southern China Sea (Marine Biological Museum, Chinese Academy of Sciences). Compared with our *O. exigua* specimens, the southern samples identified in Liao (2004) exhibit more sparsely distributed and elongated club‐shaped spines on the disk, less densely covered radial shields with clearly visible outlines, more slender and transparent arm spines, and distinctive longitudinal black and white stripes on their dorsal arm plates. Based on morphological characteristics, the southern specimens should be assigned to *Ophiothrix (Ophiothrix) ciliaris* Lamarck, 1816 (Liao, 2004).

## AUTHOR CONTRIBUTIONS

**Qian Zhang:** Resources (equal); writing – original draft (supporting). **Xuying Hu:** Resources (equal). **Zongjing Deng:** Resources (equal). **Yixuan Li:** Writing – review and editing (equal). **Yue Dong:** Resources (equal); writing – review and editing (equal). **Chen Han:** Resources (equal). **Xiaoqi Zeng:** Writing – review and editing (equal). **Ning Xiao:** Resources (equal). **Xuelei Zhang:** Conceptualization (equal). **Qinzeng Xu:** Conceptualization (equal); funding acquisition (supporting); writing – review and editing (equal).

## CONFLICT OF INTEREST STATEMENT

The authors declare that they have no known competing financial interests or personal relationships that could have appeared to influence the work reported in this paper.

## Supporting information


Figure S1.

Table S1.

## Data Availability

The sequences newly generated are deposited in GenBank (accession numbers OR782206‐OR782277 & OR984206‐OR984213for *COI*, OR780679‐OR780743 & PP001162‐PP001168 for *NAD4*, OR784232‐OR784298 & PP001711‐PP001718 for *ITS2*, and OR775595‐OR775657 & OR988137‐OR988143 for *18S*).
