# Supplementary material for: Population genetics and evolutionary history of the intertidal brittle star Ophiothrix (Ophiothrix) exigua in the northern China Sea
Source: Ecol Evol. 2024 Sep 16;14(9):e70284. doi: 10.1002/ece3.70284 (PMC11405633; doi:10.1002/ece3.70284)
Supplement: Supplementary file 1 — Figure S1. [file ECE3-14-e70284-s002.docx]

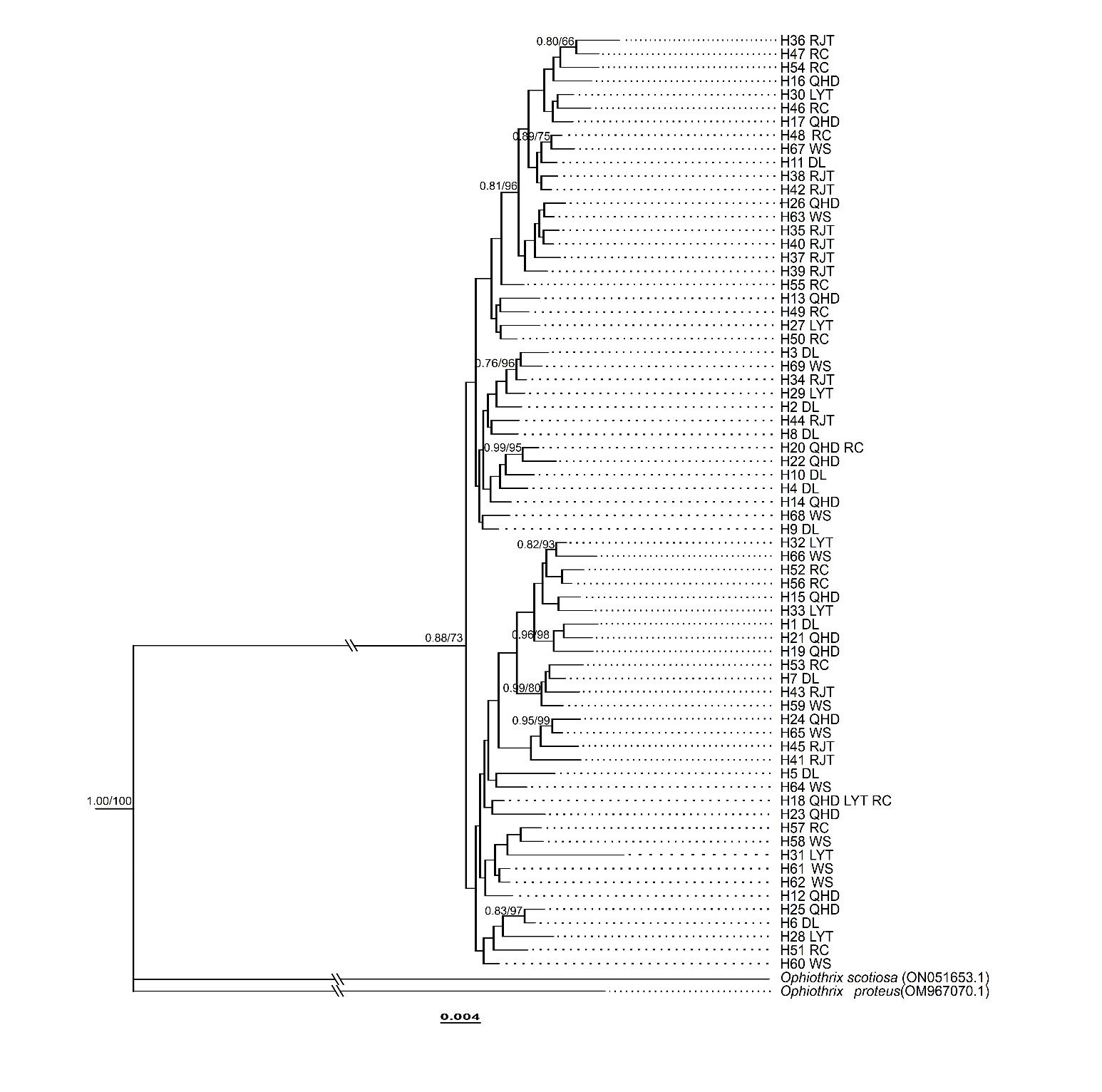


**Figure S1** Phylogenetic tree of *Ophiothrix exigua* haplotypes based on concatenated *COI*-*NAD4*-*ITS2* sequences. BI and ML yielded identical topologies, so only the Bayesian tree is shown. Posterior probability and ML bootstrap values are indicated near the branches. (Branch length of outgroup concealed).
Abbreviations: QHD, Qinhuangdao; DL, Dalian; RC, Rongcheng; WS, May Fourth Square; LYT, Langyatai; RJT, Renjiatai.
