# Supplementary material for: Population genetics and evolutionary history of the intertidal brittle star Ophiothrix (Ophiothrix) exigua in the northern China Sea
Source: Ecol Evol. 2024 Sep 16;14(9):e70284. doi: 10.1002/ece3.70284 (PMC11405633; doi:10.1002/ece3.70284)
Supplement: Supplementary file 2 — Table S1. [file ECE3-14-e70284-s001.docx]

**Table S1.** Main morphological variation of *Ophiothrix (Ophiothrix) exigua*.

Abbreviations: QHD, Qinhuangdao; DL, Dalian; RC, Rongcheng; WS, May Fourth Square; LYT, Langyatai; RJT, Renjiatai.

| Locality | Color | Patterning of arms |
| --- | --- | --- |
| QHD | Brown | annulations on the arms |
| DL | Dark brown | / |
| RC | Dark grey | / |
| WS | Dark grey | annulations on the arms |
| LYT | Grey | annulations on the arms |
| RJT | Grey | / |
